# Hepatobiliary and pancreatic manifestations in inflammatory bowel diseases: a referral center study

**DOI:** 10.1186/s12876-019-0967-3

**Published:** 2019-04-03

**Authors:** Fotios S. Fousekis, Konstantinos H. Katsanos, Vasileios I. Theopistos, Gerasimos Baltayiannis, Maria Kosmidou, Georgios Glantzounis, Leonidas Christou, Epameinondas V. Tsianos, Dimitrios K. Christodoulou

**Affiliations:** 1 Department of Gastroenterology and Hepatology, Medical school and University of Ioannina, Ioannina, Greece; 2 Department of Internal Medicine, Medical school and University of Ioannina, Ioannina, Greece; 3 Department of Surgery, Medical school and University of Ioannina, Ioannina, Greece

**Keywords:** Inflammatory bowel disease, Hepatotoxicity, Acute pancreatitis, Hepatobiliary manifestations, Pancreatic manifestations, Fatty liver, Immunomodulators

## Abstract

**Background:**

Hepatobiliary and pancreatic manifestations have been reported in patients with Crohn’s disease or ulcerative colitis. Our aim was to describe the prevalence of hepatobiliary and pancreatic manifestations in inflammatory bowel disease and their association with the disease itself and the medications used.

**Methods:**

Data were retrospectively extracted from the clinical records of patients followed up at our tertiary IBD referral Center.

**Results:**

Our study included 602 IBD patients, with liver function tests at regular intervals. The mean follow-up was 5.8 years (Std. Dev.: 6.72). Abdominal imaging examinations were present in 220 patients and revealed findings from the liver, biliary tract and pancreas in 55% of examined patients (120/220). The most frequent findings or manifestations from the liver, biliary tract and pancreas were fatty liver (20%, 44/220), cholelithiasis (14.5%, 32/220) and acute pancreatitis (0.6%, 4/602), respectively. There were 7 patients with primary sclerosing cholangitis. Regarding hepatitis viruses, one-third of the patients had been tested for hepatitis B and C. 5% (12/225) of them had positive hepatitis B surface antigen and 13.4% had past infection with hepatitis B virus (positive anti-HBcore). In addition, most of the patients were not immune against hepatitis B (negative anti-HBs), while 3% of patients were anti-HCV positive and only one patient had active hepatitis C. Furthermore, 24 patients had drug-related side effects from the liver and pancreas. The side effects included 21 cases of hepatotoxicity and 3 cases of acute pancreatitis. Moreover, there were two cases of HBV reactivation and one case of chronic hepatitis C, which were successfully treated.

**Conclusion:**

In our study, approximately one out of four patients had some kind by a hepatobiliary or pancreatic manifestation. Therefore, it is essential to monitor liver function at regular intervals and differential diagnosis should range from benign diseases and various drug related side effects to severe disorders, such as primary sclerosing cholangitis.

## Background

Ulcerative colitis and Crohn’s disease are inflammatory diseases which affect the gastrointestinal tract. However, both disorders can also involve other organ systems as well. It is not unusual for patients with IBD to have manifestations from the liver, pancreas, gallbladder and biliary tree. The strongest associated disease with IBD is primary sclerosing cholangitis (PSC). PSC is an idiopathic chronic progressive disease of the biliary tree and can cause stenosis and destruction of extra- and intrahepatic bile ducts. It is estimated that 5% of patients with UC develop PSC and up to 80–90% of patients with PSC have UC [1]. Other manifestations from the biliary tree in IBD patients include cholangitis and cholangiocarcinoma, mainly as complications of PSC [2]. The gallbladder is an organ that may be affected by IBD. Cholelithiasis is more common in patients with CD than in general population and patients with CD have a two-fold increased risk of gallstones. On the other hand, patients with UC do not have any additional risk [3]. The main independent risk factors for the development of gallstones are ileo-colonic CD location, the extent of ileal resection (> 30 cm), disease duration (> 15 years) and multiple or prolonged total parenteral nutrition treatments [4]. Hepatic manifestations in IBD vary and range from benign disorders, such as fatty liver to end-stage hepatic failure as a complication of PSC or primary biliary cirrhosis. Non-alcoholic fatty liver disease (NAFLD) refers to the accumulation of fat in hepatocytes without alcohol use. Hepatic steatosis can be diagnosed either by imaging or by histology (biopsy). Prevalence of NAFLD in IBD patients varies, ranging from 8.2 to 40% [5]. Furthermore, it seems that IBD patients develop NAFLD with fewer metabolic risk factors than general population [6]. Other hepatic manifestations in patients with IBD include liver abscess [7], granulomatous hepatitis, hepatic amyloidosis and primary biliary cirrhosis (PBC). The first three disorders are more frequent in CD, while PBC is more common in patients with UC [8].

In addition, drug-induced hepatotoxicity is common side effect of IBD treatment. More analytically, in the United Kingdom from 1991 to 1998, the incidence of hepatitis was 3.2 and 6 cases per million prescriptions for mesalazine and sulfasalazine, respectively. What is more, it seems that there is a stronger association between 5-ASA induced hepatotoxicity and rheumatoid arthritis than IBD [9]. Also, methotrexate has been associated with liver damage and it was proposed that the mechanism is dose dependent. A meta-analysis of clinical trials showed that the rate of abnormal aminotransferase serum levels [defined as up to a 2 fold elevate over the upper limit of the normal (ULN)] in patients treated with methotrexate for IBD was 1.4 per 100 person-months, while the incidence of hepatotoxicity (defined as greater than a 2 fold over ULN) was 0.9 per 100 person-months [10]. On the other hand, thiopurine induced liver damage is not dose dependent and the abnormalities in liver tests occur more frequently in the first months of therapy [11]. The incidence of thiopurine-induced liver injury is approximately 4% [12]. Anti–tumor necrosis factor (anti-TNF) biological agents rarely cause liver damage. Anti-TNF-induced liver damage may occur irrespectively of the number of infusions or injections, time and dose [13]. Additionally, patients with IBD have reduced immunogenicity due to the presence of IBD (malabsorption and malnutrition) and immunosuppressive treatment (immunomodulators and biological agents). Hence, IBD patients have an increased risk of hepatitis B reactivation or loss of immunity against hepatitis B and exacerbation of hepatitis C [14].

Acute pancreatitis and, more infrequently, chronic pancreatitis and autoimmune pancreatitis may occur in patients with IBD due to the disease itself or side effects of medication used in the treatment. The increased incidence of acute pancreatitis in Crohn’s disease can be attributed to anatomic abnormalities of the duodenum and more frequent development of cholelithiasis in Crohn’s disease as a result of ileal disease [15]. Also, acute pancreatitis is a fairly frequent side effect of azathioprine and 6-mercaptopourine use (4%) [12] and a less common side effect of 5-aminosalicylate (5-ASA) and corticosteroid treatment [16].

The aim of this retrospective study was to document the manifestations of the liver, pancreas and biliary tree in patients with Inflammatory Bowel Disease and describe their association with the disease itself and the medications used.

## Methods

In our retrospective study, we included 602 patients with IBD, who were monitored at our tertiary University Hospital from 1977 to 2016. The diagnosis of the disease was based on histopathological findings after ileocolonoscopy with biopsies. Inclusion criteria for the study were diagnosis of IBD and regular follow-up from time of diagnosis with frequent laboratory tests. Consequently, every patient who participated in this study had baseline liver function tests measured at diagnosis of IBD and at regular intervals, at least every 6 months. All patients were followed up from the time of diagnosis of IBD in our referral center. Furthermore, 220 patients underwent imaging evaluation, such as ultrasound and computed tomography, and were tested for hepatitis viruses B and C. A complete registry of all clinical, laboratory, imaging and histologic abnormalities related to liver, biliary tree and pancreas was made.

### Statistical analysis

Data were analyzed using SPSS software version 22. All clinical and pathological features were categorized as either continuous or categorical variables. Continuous variables were summarized as means and standard deviation.

## Results

The average age of patients was 39 years (Std. Dev. 17.4) at diagnosis. 57.5% of IBD patients had UC, 42.5% CD 59.8% of patients were males and 40.2% were females. The predominant symptom during diagnosis was bloody stools (38.3%). Particularly, in patients with CD, diarrhea was the most common symptom (49%), while bloody stools was the most common symptom in patients with ulcerative colitis (60%). The mean follow-up time of patients was 5.8 years (Std. Dev.: 6.72). There was no difference between liver function tests at diagnosis of disease and during last measurement **(**Table 1**).**

**Table 1:** Liver function tests of IBD patients at diagnosis and at last follow-up

Liver Function Tests	LFT at Diagnosis	LFT at last follow-up
AST (IU/L)	23.9 (Std. Dev. 20.5)	25.5 (Std. Dev. 16.1)
ALT (IU/L)	24.8 (Std. Dev. 23.1)	26.7 (Std. Dev. 27.3)
ALP (IU/L)	90 (Std. Dev. 70.1)	82.3 (Std. Dev. 65.8)
γ-GT (IU/L)	29.7 (Std. Dev. 49.3)	26.9 (Std. Dev. 30.5)

**Fig. 1:**
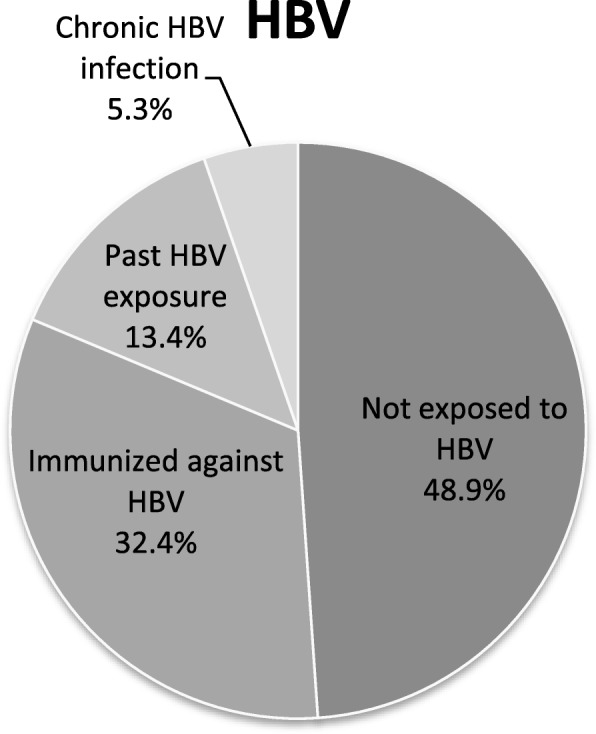
Prevalence of Hepatitis B Virus in IBD patients

Over 200 out of 602 patients were tested for antibodies against hepatitis B and C. Specifically, 5.3% (12/225) had hepatitis B (Hepatitis B surface Antigen positive, HBsAg) and 13.4% (28/208) had past HBV infection (anti-HBcore positive and HBsAg negative). Also, most of the patients were not immune against hepatitis B (67/207 anti-HBs positive, 32.4%) **(**Fig. 1**)**. With regard to hepatitis C, 3% of patients (6/201) were anti-HCV positive, but only one patient had active chronic hepatitis C (HCV-RNA positive).

Overall, 36.5% (220/602) of patients had at least one abdominal imaging evaluation. Most patients were males (134 versus 86 females), while the type of IBD was shared (107 UC, 113 CD). One hundred seventy-five patients (29.1%) underwent ultrasound which was the most frequent imaging test performed, as expected. MRCP, ERCP, CT and MRI were performed in 13, 6, 92 and 7 cases, respectively. The imaging tests revealed abnormal findings in 54.5% of examined patients (120 patients) **(**Table 2**)**. The findings ranged from benign and innocent lesions, such as liver cyst and hemangioma to malignant and severe diseases such as cholangiocarcinoma and primary sclerosing cholangitis. Most of the findings were from the liver (74 patients) and the most frequent hepatic manifestation (20%) was fatty liver. Most of the patients with fatty liver were male (28 males versus 16 females) and had ulcerative colitis (28 UC, 16 CD). Hemangioma (11 cases), cysts (14 cases), ischemic hepatitis (1 case), multiple liver abscesses (1 case), primary biliary cirrhosis (1 case), portal vein thrombosis (1 case), fibrosis (1 case) and cirrhosis (1 case) were the other liver diseases. In the case of patient with portal vein thrombosis, the risk factors for portal vein thrombosis, such as myeloproliferative disorders, protein S and C deficiency and antiphospholipid syndrome, were investigated and were negative. So, IBD was implicated as the main risk factor. The patient had moderate ulcerative colitis. The patient with liver abscesses had Crohn’s disease, while the patient with primary biliary cirrhosis had ulcerative colitis. The most frequent manifestation of the biliary tract was cholelithiasis. The patients with cholelithiasis more frequently had Crohn’s disease (20 CD, 12 UC). Cholecystitis, PSC, polyps in the gallbladder and cholangiocarcinoma were the other findings from the biliary tract **(**Table 2**).** From the pancreatic manifestations, the most common was acute pancreatitis (4 cases). All patients with acute pancreatitis had Crohn’s disease. In 3 out of 4 cases, acute pancreatitis was caused by medication, while in one case, the cause was gallstones. Two cases of drug-induced pancreatitis were caused by azathioprine and one case was caused by mesalamine. Drug-induced pancreatitis was established as diagnosis, when there was a reasonable temporal sequence between AP development and administration of the drug and withdrawal of drug causes clinical improvement. Re-exposure (re-challenge) was not used in any patient. All patients with drug-induced pancreatitis were young (17, 21 and 36 years of age), acute pancreatitis was mild and all patients were treated with discontinuation of the suspect medication and adequate intravenous fluid resuscitation. In addition, azathioprine caused two cases of significantly elevated amylase and after azathioprine withdrawal, amylase was normalized.

**Table 2:** Presentation of characteristics (age, sex, localization) of IBD patients at diagnosis and the frequency of suggestive imaging findings, overall and in ulcerative colitis and Crohn’s disease separately

	Total number of IBD patients	Patients with Ulcerative colitis		Patients with Crohn’s disease	
Severity at diagnosis of IBD		According to MAYO score 48% (166/346) Mild 29% (100/346) Moderate 23% (80/346) Severe		According to CDAI score 27% (69/256) Mild 41% (105/256) Moderate 32% (82/256) Severe	
Localization		24% (83/346) Proctitis 41% (142/346) Left sided colitis 35% (121/346) Pancolitis		29% (74/256) Ileitis 38% (97/256) Colitis 33% (85/256) Ileitis and colitis	
Age at diagnosis	39 (Std. Dev. 17.4)	42 (Std. Dev. 18.9)		35 (Std. Dev. 16.3)	
Sex (Male/Female)	360 (60%)/ 242 (40%)	202 (58%) /144 (42%)		162 (63%) /94 (37%)	
Fatty liver	44 (20%, 44/220)	28	(26.2%, 28/107)	16	(14.2%, 16/113)
Hemangioma	11 (5%, 11/220)	7	(6.5%, 7/107)	4	(3.5%, 4/113)
Cyst	14 (6.3%, 14/220)	8	(7.5%, 8/107)	6	(5.3%, 6/113)
Multiple liver abscesses	1 (0.4%, 1/220)	0	(0%)	1	(0.9%, 1/113)
Fibrosis	1 (0.4%, 1/220)	0	(0%)	1	(0.9%, 1/113)
Cirrhosis	1 (0.4%, 1/220)	0	(0%)	1	(0.9% 1/113)
Portal vein thrombosis	1 (0.4%, 1/220)	1	(0.9%, 1/107)	0	(0%)
Cholelithiasis	32 (14.5%, 32/220)	12	(11.2%, 12/107)	20	(17.7%, 20/113)
Primary sclerosing cholangitis	7 (3.2%, 7/220)	2	(1.9%, 2/107)	5	(4.4%, 5/113)
Cholecystitis	7 (3.2%, 7/220)	3	(2.8%, 3/107)	4	(3.5%, 4/113)
Polyps in the gallbladder	5 (2.3%, 5/220)	4	(3.7%, 4/107)	1	(0.9%, 1/113)
Cholangiocarcinoma	1 (0.4%, 1/220)	1	(0.9%, 1/107)	0	(0%)
Acute pancreatitis	4 (1.8%, 4/220)	0	(0%)	4	(3.5%, 4/113)
Fatty pancreas	2 (0.9%, 2/220)	1	(0.9%, 1/107)	0	(0%)
Chronic pancreatitis	1 (0.4%, 1/220)	1	(0.9%, 1/107)	0	(0%)

### Drug-induced hepatotoxicity

Overall, 76% of patients in our study received 5-ASA, 68.1% corticosteroids, 31.5% azathioprine, 10.7% methotrexate, 15.8% infliximab and 4.5% adalimumab at some point during their therapy. The mean duration of methotrexate administration was 69.2 weeks (Std. Dev. 71.6) and the mean cumulative dose was 1287 mg, while the mean time of azathioprine administration was 597 days (Std. Dev. 522). In 21 cases, IBD treatment caused drug induced liver injury (DILI). DILI was defined when other causes of liver injury, such as viral hepatitis and PSC, had been excluded and withdrawal of drug caused laboratory improvement. Also, we used the Roussel Uclaf Causality Assessment Method (RUCAM) for the definition of DILI. The mean age of patients with hepatotoxicity was 37 years (17 to 72) and the majority of these patients were males (14 males versus 7 females). None of them had preexisting liver disease. The drug-induced hepatotoxicity was caused by methotrexate, azathioprine and mesalamine. Hepatotoxicity occurred in the first weeks of treatment. After drug withdrawal, the liver function tests returned to normal **(**Table 3**).**

**Table 3:** Information about drug-induced hepatotoxicity. The table demonstrates the mean duration of drug administration, the number of cases with hepatotoxicity for each drug and the mean value (range) of liver enzymes during toxicity

Drug	Duration of medication	Overaall number of cases	AST (U/L)	ALT (U/L/)	ALP (U/L)	GGT (U/L)	Cases with ALT > 3	Cases with ALT > 5
Methotrexate	10 weeks (4–20)	9	77 (30–128)	170 (62–382)	100 (43–234)	63 (23–152)	1	
Azathioprine	5.5 weeks (3–9)	9	383 (69–1165)	280 (113–594)	260 (43–613)	768 (38–2143)		3
Mesalamine	15 weeks (13–17)	3	63 (51–75)	97 (71–123)	83 (78–88)	45 (42–48)		

**Fig. 2:**
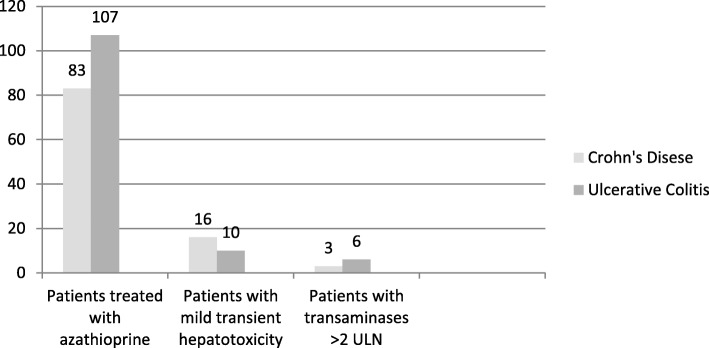
Presentation of patients who were treated with azathioprine and presented mild (< 2 ULN) or severe (> 2 ULN) hepatotoxicity

Particularly, according to R factor for liver injury, in 8 of the 9 AZA-hepatotoxicity cases hepatotoxicity liver injury was observed (R > 5) and in only one case the cause of liver injury was mixed (R: 2–5). Furthermore, there were 22 cases of mild transient elevation of transaminases [definite as up to a 2-fold elevate over the upper limit of the normal range (ULN)]. Mild elevations of transaminases occurred approximately 30 weeks after inception of drug administration **(**Fig. 2**).** From patients with hepatotoxicity, there were 3 patients with ALT > 5 ULN and four patients with ALT > 3 ULN, while there was not any patient with Hy’s Law. Specifically, 10 patients suffered from UC, while 16 patients had CD.

There were two cases of HBV reactivation in patients with CD who were treated with azathioprine. They had inactive hepatitis B with normal liver function tests prior to the administration of azathioprine B and they did not receive any chemoprophylaxis. In both cases, at reactivation of HBV, transaminases were more than 10-fold of upper limit of the normal range (ULN), and HBV-DNA was > 20,000 IU/mL. In one case, reactivation occurred 8 months after the start of azathioprine administration and 2 months after the start of 8 mg of methylprednisolone. The patient received both drugs for 2 months. After HBV reactivation, methylprednisolone was discontinued, the patient was successfully treated with entecavir, achieving both biochemical and virological response (with negative HBV-DNA). In the other case, the reactivation of HBV occurred 50 days after azathioprine administration, with HBV-DNA: 10,000 IU/mL, ALT: 94 U/L and AST 83 U/L. The patient was treated with tenofovir and also achieved biochemical and virological response. Additionally, a female patient with UC, who was treated with azathioprine for 2 years and suffered from chronic hepatitis C, developed elevated transaminases more than 2 ULN, while HCV-RNA was positive. Eventually, she was successfully treated with ledispavir 90 mg and sofosbuvir 400 mg for 12 weeks, achieving sustained virological response after treatment. Liver biochemical tests returned to normal levels after HCV therapy.

### Primary Sclerosing cholangitis

Among 602 patients with IBD, there were 7 patients who were diagnosed with large duct primary sclerosing cholangitis. Surprisingly, 5 of these patients suffered from Crohn’s disease and only two from ulcerative colitis. Also, five were males and two females. Apart from one case, in which IBD was diagnosed 7 years after diagnosis of PSC, in the other 6 cases, PSC and IBD were diagnosed at the same period. The average age of PSC diagnosis was 32.8 years old (17 to 56) and the mean follow-up of PSC was 10 years (1 to 19 years). One patient underwent liver transplantation because she developed liver failure 12 years after diagnosis of PSC, and another patient developed cirrhosis after 7 years from diagnosis. All patients were treated with ursodeoxycholic acid, while gallstones were found in two of the seven patients with PSC. Table 4 shows the liver function tests on the last visit to the hospital **(**Table 4**)**.

**Table 4:** Presentation of patients with primary sclerosing cholangitis, their liver function tests on the last visit to the hospital and their outcomes

Gender of PSC patient	Age at diagnosis(years)	Type of IBD	AST (U/L)	ALT (U/L)	ALP (U/L)	GGT (U/L)	TBL (mg/dL)	Duration PSC (years)	Outcome
1.Male	32	CD	31	49	53	30	1	14	Slow progression
2.Male	48	UC	33	62	103	132	0.8	9	Slow progression
3.Female	51	CD	133	190	716	171	3.7	9	Before liver transplantation
4.Male	17	CD	14	19	50	33	0.7	1	Slow progression
5.Female	55	CD	41	56	43	15	0.9	19	Slow progression
6.Male	27	CD	18	15	61	21	0.9	5	Slow progression
7.Male	68	UC	97	58	92	41	0.7	12	Cirrhosis

## Discussion

Over 50% (120/220) of patients undergoing imaging evaluation presented findings or manifestations from the liver, biliary tract and pancreas. Fatty liver was the most frequent finding (20%). Imaging evaluation was performed, when there was an indication, such abnormal biochemical liver function tests, and this is one of the limitations of our study. This prevalence of fatty liver in our patients with inflammatory bowel disease was lower than what Bargiggia et al. have found (37%) [5]. The increased prevalence of liver steatosis in IBD can be explained by improved IBD treatment, with most of the IBD patients not being malnourished and usually having normal or increased weight. Furthermore, IBD therapy includes medications such as corticosteroids and methotrexate that have been associated with liver steatosis development [17]. Unfortunately, there was no accurate information about alcohol consumption, BMI and factors-related with metabolic syndrome of our patients. Despite that, most patients did not consume alcohol at all and the remainder were barely social drinkers. In addition most of them had normal BMI. So it is unlikely that these factors had played a significant role on steatosis.

Concerning the biliary tract, cholelithiasis was the most frequent finding as expected. In our study, the prevalence of cholelithiasis was 14.5% in IBD patients and 18% specifically in patients with Crohn’s disease, with a highest number of males having cholelithiasis (18 males versus 14 females). The prevalence of cholelithiasis was similar in comparison with other studies, as the prevalence of cholelithiasis in CD ranges from 11 to 34%, while in general population, it ranges from 5.5 to 15% [3]. Concerning PSC, the prevalence of PSC in IBD patients and particularly in patients with UC was lower than reported in the literature (UC 0.7% and CD 2.5% versus UC 5% and CD 3%) [18]. The decreased incidence of PSC in our retrospective study may be due to limited use of MRCP in the first two decades of retrospective study. Our tertiary center has started to use MRCP since 1993. Also, the males with PSC were more than the females with PSC and the average age of patients with PSC was 32.8 years. It has been described that PSC occurs in middle age with a 2:1 male predominance [19]. Furthermore, gallstones were found in 28% (2/7) of patients with PSC, as cholelithiasis is a common finding in patients with PSC, and approximately 25% of PSC patients have gallstones [20].

Regarding the pancreas, acute pancreatitis was the most common manifestation and the cumulative incidence rate was 0.76%, with 5.8 years mean follow-up period. All cases occurred in patients with CD. The cumulative incidence rate of acute pancreatitis in patients with CD was 2%. In another study, the incidence was greater, but the follow-up time was longer (cumulative incidence 1.6% with follow-up for 14 years) [21]. In a Danish follow-up study, the risk of acute pancreatitis was four fold in patients with CD [22] and in a retrospective study of 852 patients with CD and a follow-up period of 10 years, the described frequency of acute pancreatitis was 1.4% [23]. Drug-induced acute pancreatitis is one of the most severe complications of IBD medication and many medications, such as thiopurines, corticosteroids, metronidazole and biological agents, have been implicated. In our study, in 3 out of 4 cases, acute pancreatitis was caused by medication. The drug-induced pancreatitis occurred a few days after the beginning of therapy (for azathioprine cases after 10 and 25 days and for mesalazine case after 28 days). The delay between drug introduction and acute pancreatitis was similar to that of the literature described [21]. Additionally, it is worth mentioning that the differential diagnosis of causes of acute pancreatitis in IBD patients should include DIPI, biliary pancreatitis, auto-immune pancreatitis and duodenal involvement.

Viral hepatitis can be a major problem in IBD patients and all IBD patients should be tested for viral hepatitis. In our study, approximately 35% of patients had evidence of their immunization for hepatitis B and C status in their medical records. Present and/or past HBV infectious was found in 24.1% of patients with UC and in 14.2% of patients with CD. Active HBV infection was found in 11 patients with UC, but only in one patient with CD. Also, past HCV infections were found in 5 patients with UC [anti-HCV positive and HCV RNA negative (undetectable), 6.2%], while one patient with UC had chronic HCV infection (anti-HCV positive and HCV RNA positive). This prevalence is very high and it seems that in the past HBV and HCV screening was probably performed selectively, when HBV and HCV infections were suspected. In a Spanish multicenter study with 2076 IBD patients, present and/or past HBV and HCV infections were found in 9.7% of IBD patients [24]. In patients, who were tested for anti-HBs (antibodies against HBsAg), only 32.4% proved positive. Furthermore, another retrospective study reported low proportion (51%) of immunity against HBV in patients with IBD [25]. The low proportion of IBD patients with immunity against HBV is partly due to the reduced immunogenicity, because of disease course and immunosuppressive treatment. Apart from that it has been also reported that IBD patients have poorer response to HBV vaccination than general population (< 50% versus 95%) [26] and a high proportion of IBD patients with protective anti-HBs titers after vaccination lose them over time [27]. The cases with HBV reactivation show that we should screen patients who will receive immunosuppressive therapy even when the liver function tests are normal (actually in all cases). All HBsAg-positive should receive entecavir or tenofovir alafenamide fumarate or tenofovir disoproxil fumarate as treatment or prophylaxis, while in patients with past HBV infection (HBsAg negative and anti-HBc positive), prophylaxis is not routinely recommended. In these patients, HBsAg and/or HBV-DNA should be monitored every 1 to 3 months during and after immunosuppressive therapy, and in case of seroconversion to positive HBsAg or detectable HBV-DNA, treatment with entecavir or tenofovir alafenamide fumarate or tenofovir disoproxil fumarate should begin immediately [28, 29]. In one of our cases with HBV reactivation, the patient was treated with simultaneous immunosuppressants (corticosteroid and azathioprine). A multicenter retrospective study reported that treatment with two or more immunosuppressants is an independent factor for HBV reactivation (OR 8.75; 95% CI 1.16 to 65.66) [30].

Methotrexate and azathioprine were the drugs administered in most cases of hepatotoxicity. Approximately 15% (1.1 per person-months) of patients who received methotrexate had liver toxicity. Methotrexate is well-known to cause hepatotoxicity, and according to a meta-analysis, the pooled incidence rate of abnormal transaminase levels [definite as up to a 2-fold elevate over the upper limit of the normal range (ULN)] in IBD patients treated with methotrexate is 1.4 per 100 person-months, while the rate of hepatotoxicity (> 2 ULN) is 0.9 per 100 person-months. Also, the rate of withdrawal from treatment due to liver injury is 0.8 per 100-person months [10]. In our study, 4.6% (9/192) of patients who received azathioprine had liver injury (1 per 35 person-years) and the incidence was similar to the literature data. Specifically, the incidence of AZA-liver injury varies and ranges from 3% (in retrospective studies) to 10% (in prospective studies) [31, 32]. In addition, hepatotoxicity from AZA was dose independent and occurred only few weeks after administration of AZA, while many patients received AZA for more than 1 year without manifesting liver injury. AZA-induced DILI was dose independent. The liver toxicity from 5-ASA agents was mild. After drug withdrawal, liver function tests normalized. We suggest discontinuing azathioprine in case of persistent elevation of aminotransferases > 3 ULN. Alternative approaches include the change to 6-mercaptopurine, which may be better tolerated, or the combination of allopurinol 100 mg daily with low dose azathioprine (approximately 30% of the initial or regular dose). With such an approach, therapeutic levels of the metabolite 6-thioguanine can be achieved, with concomitant decrease of the levels of hepatotoxic metabolites of azathioprine (6-methylmercaptopurine).

## Conclusion

Hepatobiliary and pancreatic manifestations in IBD are frequent and their range is wide. In our study, one out of four patients presented some kind of hepatobiliary or pancreatic manifestation related to the disease itself or medications. Hence, monitoring liver function in patients with IBD at regular intervals is essential and the differential diagnosis should include from side effects of therapy, and common and benign diseases, such as fatty liver, to rare and chronic diseases such as primary sclerosing cholangitis. Furthermore, we should not forget that IBD patients should be screened for viral hepatitis B and C markers and immunized against hepatitis B.

## Abbreviations

CD: Crohn’s disease
HBV: Hepatitis B virus
HCV: Hepatitis C virus
IBD: Inflammatory Bowel Disease
NAFLD: Non-alcoholic Liver Disease
PSC: Primary sclerosing cholangitis
UC: Ulcerative colitis
